# Imaging Neuroscience: The First Three Years—Editorial

**DOI:** 10.1162/IMAG.a.1392

**Published:** 2026-10-08

**Authors:** Stephen M. Smith

**Affiliations:** OxCIN (FMRIB), University of Oxford, Oxford, United Kingdom

## Abstract

In April 2023, the editors of *NeuroImage* and *NeuroImage:Reports* resigned, and a month later launched *Imaging Neuroscience*. *NeuroImage* had been the leading journal in our field, but its publication fee had reached a level that many considered unethical and unsustainable. We therefore launched *Imaging Neuroscience* with MIT Press, with the goal of replacing *NeuroImage* as our field’s leading journal, and with greater control by the academic community over publication fees and adoption of modern and ethical publishing practices. Three-and-a-half years later, as I step down as Editor-in-Chief, this editorial gives a brief summary of how things are going. The bottom line is: very well indeed!

## 1 Recap of All Editors Leaving *Neuroimage*

*NeuroImage* was launched in 1992, and by 2022 was publishing almost 1,000 papers per year. By 2020, its publisher, Elsevier, had been persuaded to make the journal fully open access, but with a high article processing charge (APC) of $3,000. Looking ahead, it was clear that a continued focus on profits would put unrelenting upward pressure on the APC, which is borne out by the fact that it has since risen to $3,540. Many were concerned that the excessive level of profit implied by these fees, well entrenched in for-profit academic publishing, was unethical and unsustainable.

In 2022, all 43 *NeuroImage* editors formally requested that Elsevier reduce the APC to below $2,000. Elsevier refused, and as a result (and with the support of all previous *NeuroImage* Editors-in-Chief), all editors of *NeuroImage* and *NeuroImage:Reports* resigned. For more detail on the historical background and the rationale for the journal transition, see our original editorial.^1^

## 2 Starting *Imaging Neuroscience*

In April 2023, we publicly announced our resignation and the forthcoming launch of *Imaging Neuroscience*, which began accepting submissions just a few weeks later. Support from people in our field, and beyond it, was hugely positive. *Imaging Neuroscience* is published by MIT Press, a highly respected non-profit publisher. The team at MIT Press did an amazing job of getting the journal running very professionally but incredibly quickly, with the first papers appearing in August. This rapidity was largely due to the talented people at MIT Press, but also in part because several authors took the courageous decision to transfer submissions already under review at *NeuroImage*.

There were of course many discussions among editors about all aspects of this, though not always in the most expected ways: for example, it took significantly more discussion (and multiple voting spreadsheets) to decide on the new journal name than it did to choose the new publisher! Although at *NeuroImage* the editors had been paid stipends, we all agreed not to take stipends at the new journal, at least for now, to help minimize the APC. The APC was initially set at $1,600 (now $1,400), with full waivers for authors from countries with low levels of research funding.

The non-profit nature of MIT Press is not just about minimizing the APC—it reflects a completely different underlying philosophy about why they are in this business. That difference is apparent in almost every interaction: discussions with MIT Press focus straightforwardly on what is best for the journal and its community, without a need to balance those considerations against maximizing profit. But more specifically, the combination of serious professionalism with flexibility and lightweight decision-making has made our job as editors immeasurably more rewarding. There are many unseen aspects of setting up a new journal that would have been really painful, and much slower, if we editors had attempted to build a new journal without the involvement of a professional publisher.

In addition to 44 Senior and Handling Editors, we have an Editorial Board of more than 60 academics in our field. This provides a wider pool of expertise and experience to contribute to strategic planning, as well as being a group of respected and enthusiastic reviewers. The editorial team comprises individuals with a wide range of specialties, reflecting the multidisciplinary nature of our field. Beyond expertise, when constructing the editorial team, we take seriously the importance of achieving diversity across multiple dimensions.

## 3 How Are Things Going?

When we announced the new journal, we had no way of knowing whether this would actually work. It is one thing for a community to express strong support for the principles behind a new journal; it is another for researchers to entrust their best work to a journal with no track record, no citation metrics, and of course no published papers. The speed with which authors did exactly that was probably the most important and gratifying part of the whole transition.

So we are really pleased with how quickly and robustly the journal has become established, both in terms of the quantity and quality of papers. There is clear evidence that we, the brain imaging community, have gotten fully behind the idea of a non-profit, open-access, high-quality journal; in just over three years, almost 2,900 papers have been submitted, and 1,110 published.

Editors’ quality thresholds for acceptance are the same as those we used to apply at *NeuroImage*. The percentages of the different editorial decisions at Imaging Neuroscience in 2025 were: accept = 47, reject-resubmit = 15, reject-after-review = 12, and triage = 26. One unexpected observation by our editors is that the overall quality of submissions is, on average, higher than we used to receive at *NeuroImage*. The average times for processing submissions are: 67 calendar days from submission to the first major revision decision; 7 working days from acceptance to sending to production; 2 working days from sending to production to the early-view paper appearing online; and 16 working days from sending to production to the final version appearing online (including the time taken for authors to return proofs).

What are the biggest ongoing challenges? Probably the most important right now is finding people willing to provide timely reviews. We aim to obtain at least three reviews for each paper, because we believe this is normally important for a reliable review process. Our editors, therefore, sometimes have to invite large numbers of reviewers—and chase quite a few reviews—to achieve this. It can be hard work! So, a plea to all authors (i.e., potential reviewers)—please agree to provide timely reviews when you can, and when you cannot, please do suggest alternative reviewers. We are currently thinking of ways to better engage early career reviewers—watch this space.

Financially, the journal is secure. The economies of scale resulting from the healthy number of papers being published enabled us to reduce the APC to $1,400 in 2025. This low APC is possible despite the fact that the quality of support from MIT Press is amazing, including having a superb Journal Manager and extremely high-quality paper production. This has been a major improvement on our previous experiences, with authors at *Imaging Neuroscience* much happier with the proofing and production process.

Finally, for me, an important lesson from the past three years is that academic communities have more power over publishing than we sometimes assume. A journal is ultimately not its name, its impact factor, or its publisher. Its value comes from the researchers who submit their work, the reviewers who give their expertise and time, and the editors who make strategic and editorial decisions. Those are the people who create a scientific journal, and those people can, when necessary, vote with their feet. If those people collectively decide that a different publishing model is needed, they can create one.

## 4 A New Editor-in-Chief!

I am delighted to announce that, as of September 2026, Shella Keilholz takes over as the new Editor-in-Chief of *Imaging Neuroscience*. Her selection was the result of a thorough and transparent process for choosing a new EiC, which is described in the journal’s Guide for Authors.

It was a genuine honour to serve as the EiC of *Imaging Neuroscience*, following 1.5 years as *NeuroImage* EiC, and 10 years as a Handling/Senior Editor before that. The time is right for me to step back. I am genuinely sad to be leaving the journal, but while I will miss this, I am even more happy that Shella is taking over—she will be truly excellent. I look forward to seeing where she and the rest of the team take *Imaging Neuroscience* next.

## Footnotes

1
https://doi.org/10.1162/imag_e_00007

